# Two new species of the genus *Dahlica* Enderlein (Lepidoptera, Psychidae) from Korea

**DOI:** 10.3897/zookeys.733.20793

**Published:** 2018-01-26

**Authors:** Seung Jin Roh, Bong-Woo Lee, Bong-Kyu Byun

**Affiliations:** 1 Division of Forest Biodiversity, Korea National Arboretum, Pocheon, South Korea; 2 Department of Biological Science and Biotechnology, Hannam University, Daejeon, South Korea

**Keywords:** bagworms, DNA barcode, Naryciinae, new species, Psychidae

## Abstract

The genus *Dahlica* Enderlein, 1912 is reported for the first time from Korea with two new species: Dahlica (Dahlica) somae Roh & Byun, **sp. n.** and Dahlica (Dahlica) ochrostigma Roh & Byun, **sp. n.** Adults and genitalia are illustrated, and DNA barcodes for precise identification of the species are also provided.

## Introduction

The family Psychidae is a medium-sized family of moths consisting of 241 named genera and 1,350 species (Sobczyk 2011; van Nieukerken et al. 2011). Phylogenetically, Psychidae belong to the oldest clades of the suborder Ditrysia in the order Lepidoptera, and have usually been placed in the superfamily Tineoidea (Davis and Robinson 1998), with Eriocottidae, Tineidae, Meessiidae, and Dryadaulidae as phylogenetically allied groups (Mutanen et al. 2010; Regier et al. 2015). Most species of Psychidae produce characteristic cases or bags at different larval stages (Sugimoto 2009a, 2009b), which gives rise to their common name, bagworms. Parthenogenesis is known in several species of the genus *Dahlica* Enderlein, 1912 in the Naryciinae (Grapputo et al. 2005; Elzinga et al. 2013). Identification of these species and classification of the females based on morphological and ecological characters alone is difficult (Grapputo et al. 2005; Elzinga et al. 2013). In Korea, Roh et al. (2016) reviewed the nine known species including a new species, *Psyche yeongwolensis* Byun & Roh, 2016 and recorded a species new for the country, *Proutia maculatella* Saigusa & Sugimoto, 2014. Later, Roh and Byun (2016) recorded *Ceratosticha leptodeta* Meyrick, 1935 new for Korea. Recently, three more species were reported: *Bacotia sakabei* Seino, 1981, (Roh and Byun 2017a), *Bruandella niphonica* (Hori, 1926), and *Proutia nigra* Saigusa & Sugimoto, 2014 (Roh and Byun 2017b). Consequently, 13 species in total are now known from Korea.

The genus *Dahlica* was based on the type species *Dahlica larviformis* Enderlein, 1912 by Enderlein in 1912 (Sobczyk 2011). The members of *Dahlica* are superficially similar to *Siederia* Meier, 1957 (Grapputo et al. 2005), but can be distinguished from the latter by the absence of an epiphysis on the fore-tibia of the male (Herrmann 1988; Herrmann and Weidlich 1999; Rekelj et al. 2014; Arnscheid 2016), the absence of the medial cell in the fore- and hindwings, presence of accessory cells, and six veins arising from the discoidal cell of the hindwing (Rekelj and Predovnik 2014).

Meier (1958) and Sieder (1953) proposed to divide *Dahlica* in various subgenera, which were later raised to genus. Recently Arnscheid and Weidlich (2017) reviewed the five allied genera, *Dahlica*, *Siederia*, *Brevantennia* Sieder, 1953, *Postsolenobia* Meyer, 1958, and *Praesolenobia* Sieder, 1955, and decided on the basis of the venation of the male hindwings, male forewing scale morphology, presence of an epiphysis in the males, the structure of reproductive organs, and the female antennae to sank these genera again as subgenera of *Dahlica*. They diagnosed the subgenus Dahlica by the following characters: the absence of an epiphysis, presence of six veins from the hindwing discal cell, and the long female antennae, with more than eleven segments (Arnscheid and Weidlich 2017).

Females of the genus *Dahlica* are unable to fly because of their degenerate wings (Sauter and Hättenschwiler 1999). The larvae feed on moss, algae, and lichens, which are attached to walls or the bark of trees via a sac constructed of small sand particles (Sauter and Hättenschwiler 1999; Sugimoto 2009a; Arnscheid and Weidlich 2017).

In total, 42 species of the subgenus Dahlica have been reported worldwide and are distributed throughout the Palaearctic region in Europe (41 species) and Asia (one species) (Sobczyk 2011; Arnscheid and Weidlich 2017).

In this study, Dahlica (Dahlica) somae sp. n. and D. (D.) ochrostigma sp. n. are described as new species and the genus *Dahlica* is reported for the first time from Korea. All available information is presented, including the collection locations, micro-habitats, and illustrations of adults and their genitalia. DNA barcodes are also provided for precise identification of each species.

## Materials and methods

The material examined in this study is preserved in the Systematic Entomology Laboratory, Hannam University (**SEL/HNU**), Daejeon, Korea, and the Entomological Collection of the Korea National Arboretum, Pocheon, Korea (**KNAE**). Specimens were dissected and examined after mounting on slide glass; male genitalia and wing scales in 80 % glycerol solution, females in euparal solution and wing venation on dried condition. Photographs of adults and genitalia were taken using a PAXcam digital camera (PAXcam Microscope Cameras Co., Chicago, IL, USA) attached to a Carl Zeiss Axio Imager A1 microscope (Carl Zeiss Ltd., Cambridge, MA, USA).

**Figures 1–4.**
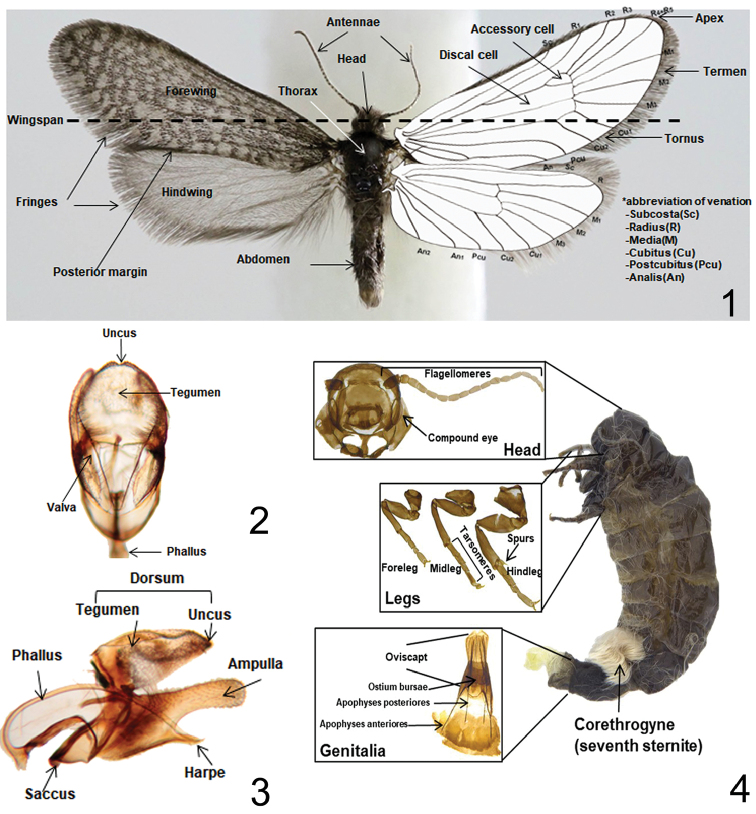
Terminology of morphological characters. **1** Male (Dierl (1964), Arnscheid and Weidlich (2017)) **2** Male genitalia, dorso-ventral part (Dierl (1964), Kristensen (2003) and Arnscheid and Weidlich (2017)) **3** Ditto, lateral part (Dierl (1964), Kristensen (2003) and Arnscheid and Weidlich (2017)) **4** Female (Arnscheid and Weidlich (2017)).

Terminology and morphological characters of the adult, wing venation, and genitalia follows Dierl (1964), Kristensen (2003), and Arnscheid and Weidlich (2017) (Figs 1–4) and the terminology for forewing scales (class 1 to 6) follows Sauter (1956). The set-up of the data matrix for morphological characters of the genus *Dahlica* follows Arnscheid (2016) (Table 2).

**Table 1.** Species with DNA barcodes and GenBank accession numbers used in this study.

Scientific name	Country	BIN number	Accession number (GenBank)	Basepair length
Dahlica (Dahlica) somae sp. n.	Korea	BOLD:ADJ8202	MF508656	658
D. (D.) somae	Korea	BOLD:ADJ8201	MF664099	658
D. (D.) somae	Korea	BOLD:ADJ8201	MF664100	658
D. (D.) ochrostigma sp. n.	Korea	BOLD:ADK4708	MF508657	658
D. (D.) ochrostigma	Korea	BOLD:ADK8063	MF664101	658
D. (D.) ochrostigma	Korea	BOLD:ADK8063	MF664102	658
D. (D.) triquetrella (Hübner)	Canada	–	KR941436	591
D. (D.) triquetrella	Switzerland	–	KX045622	658
D. (D.) triquetrella	Slovenia	–	KX045823	658
D. (D.) lichenella (Linnaeus)	Canada	–	KR941275	591
D. (D.) fennicella (Suomolainen)	Finland	–	JX307942	657
D. (D.) lazuri (Clerck)	Finland	–	JX307894	657
D. (D.) goltella Rekelj & Predovnik	Slovenia	–	KX045455	658
D. (D.) charlottae (Meier)	Finland	–	JX307874	657
D. (D.) parthenogenesis (Saigusa)	Japan	–	LC094189	665
Dahlica (Postsolenobia) juliella (Rebel)	Slovenia	–	KX047137	658
Dahlica (Siederia) listerella (Linnaeus)	Japan	–	LC094179	665
D. (S.) listerella	Austria	–	KP150244	658
D. (S.) listerella	Finland	–	KJ192386	658
D. (S.) rupicolella (Sauter)	Finland	–	KJ192382	658
Dahlica (Bevantennia) adriatica (Rebel)	Slovenia	–	KX045214	658
*Narycia emikoae* Niitsu, Jinbo & Nasu	Japan	–	LC160295	658
*Narycia duplicella* (Goeze)	Slovenia	–	KX045830	658
*N. duplicella*	Belgium	–	KC305219	658

**Table 2.** Data matrix for morphological characters (Arnscheid 2016) of *Dahlica* species in Korea.

**Species**	**Male wingspan**	**Scales (classes)**	**Hindwing venation (M_2_/M_3_)**	**Genitalia index**
D. (D.) somae	12.3–13.4 mm.	2–4	free	1.46–1.56
D. (D.) ochrostigma	9.8–11.2 mm.	1–2	short stalked	0.79–1.08

Genomic DNA was extracted from the legs of dried specimen for males and thorax parts of immersion specimen for females, preserved in 100% alcohol using a Genomic Cell/Tissue Spin Mini Kit (Mbiotech, Inc., Hanam, Korea), according to the manufacturer’s protocol. A total of six specimens were sequenced for, the 658 bp fragment of the mitochondrial cytochrome c oxidase I (COI) gene, the DNA barcode, was amplified using the primer pair LepF1 and LepR1 (Hebert et al. 2004). PCR conditions for amplification followed the manufacturer’s protocol (Platinum Taq, Invitrogen, Carlsbad City, CA, USA). Amplicons were purified using the QIAquick® PCR purification kit (QIAGEN, Inc.) and directly sequenced at Genotech Corp. (Yuseong-gu, Daejeon, Korea). Contigs were assembled using CodonCode aligner version 2.0.6 (CodonCode Co., Centerville City, MA, USA) and were aligned using MAFFT (Katoh and Toh 2008).

The new barcodes were compared to 18 DNA barcodes of the genera *Dahlica* and *Narycia* downloaded from GenBank (National Center for Biotechnology Information, USA, http://www.ncbi.nlm.nih.gov/) (Table 1). A neighbor-joining (NJ) analysis was performed with MEGA 6.0 (Tamura et al 2013) under the K2P model for nucleotide substitutions. Successful sequences were uploaded to BOLD systems (project. KNAE) and submitted to GenBank (Table 1).

## Systematic accounts

### Dahlica

Taxon classificationAnimaliaORDOFAMILIA

Enderlein, 1912

Subgenus Dahlica Enderlein, 1912
Dahlica Enderlein 1912: 264.

#### Type species.

*Dahlica larviformis* Enderlein, 1912: 264 by monotypy.

#### Key to the males of *Dahlica* in Korea

**Table:** 

1	Hindwing M2 and M3, originate at apical corner of posterior part of discoidal cell (Fig. 28), dorsum of genitalia gently curved to apical part and harpe hooked (Fig. 11)	***D. (D.) somae* sp. n.**
–	Hindwing M_3_ stalked at 1/4 M_2_, dorsum strongly arched to apical part (Fig. 29) and harpe needle shape (Fig. 18)	***D. (D.) ochrostigma* sp. n.**

### Dahlica (Dahlica) somae

Taxon classificationAnimaliaORDOFAMILIA

Roh & Byun sp. n.

http://zoobank.org/E35CEE22-4005-4581-AFAD-DEB937241716

Figs 5–12 , 24–27 , 28 , 32 , 33

#### Type material.

***Holotype*.** ♂, **Korea**: Daejeon, Mt. Heungnyongsan, 15.ii.2015, S.J. Roh & D.S. Kim, genitalia mounted on 80% glycerol solution, genitalia No. KNAESJ01, scales of forewing mounted on 80% glycerol solution, scales of forewing No. KNAESSJ01, venation of forewing No. KNAEVSJ01, DNA barcode accession No. MF508656. Deposited at SEL/HNU.

***Paratypes*.** 2♂, 1♀. **Korea**: 1♂ Daejeon, Isa-dong, 2.ii.2015, S.J. Roh, genitalia mounted on 80% glycerol solution, genitalia No. KNAESJ02, scales of forewing mounted on 80% glycerol solution, scales of forewing No. KNAESSJ02, venation of forewing No. KNAEVSJ02, DNA barcode accession No. MF664099; 1♂ Daejeon, Mt. Heungnyongsan, 6.iii.2017, S.J. Roh & D.S. Kim, genitalia mounted on 80% glycerol solution, genitalia No. KNAESJ03; 1♀ Mt. Heungnyongsan, 6.iii.2017, S.J. Roh & D.S. Kim, DNA barcode accession No. MF664100. Deposited at SEL/HNU.

#### Diagnosis.

Male of this species is superficially similar to *D. triquetrella* (Hübner, 1813), but can be distinguished by a slightly longer transtilla and a relatively short ampulla of the male genitalia (lateral aspect). This species can be readily differentiated by the veins of the male hindwing; M2 and M3 originate at the apical corner of the posterior part of the discoidal cell. Female apophyses posteriores 1.75 times longer than apophyses anteriores.

#### Description.

**Figures 5–12.**
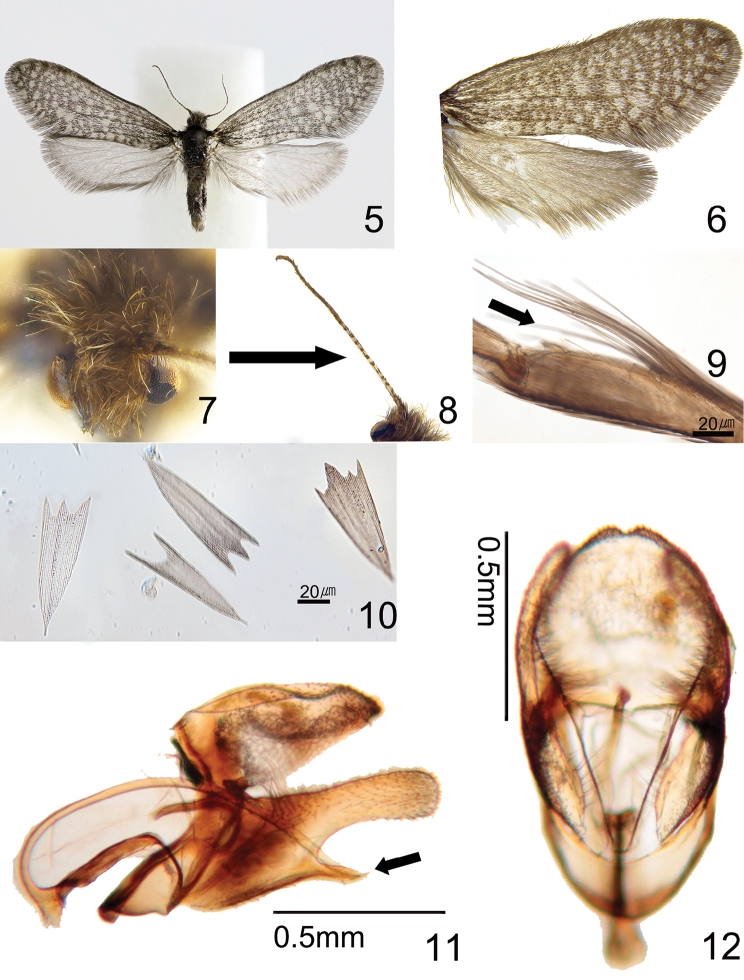
Male of Dahlica (Dahlica) somae, holotype. **5** Male **6** Close-up of rightwing- pattern **7** Head, frontal view **8** Antenna **9** Absence of foreleg-tibia **10** Scales of forewing (slide No. KNAESSJ01) **11** Genitalia (slide No. KNAESJ01), lateral view **12** Ditto, dorso-ventral view.

Adult. Male (Figs 5–12). Wingspan 12.3–13.4 mm (Table 2). Coloration and vestiture: Vertex of head roughly covered with grayish brown hairs. Thoracic notum covered with blackish brown hairs. Upper side of forewing: ground color grayish black; white spots present regularly; scales (Fig. 10) slightly narrow and evenly widened apically; apical margin usually produced into two to four laciniations (classes 2–4) (Table 2). Hindwing covered with grayish white scales; postmarginal part present with slight long shiny white hairs. Structure: head and compound eyes slightly large; ocelli absent. Antennae (Fig. 8) filiform, longer than 2/3 forewing. Forewing: slightly long and narrow; costa straight; termen shortly arched to posterior margin, discoidal cell 0.64 times as long as forewing; venation (Fig. 28) with nine veins, originating at the discoidal cell; accessory cell present; intercalary cell absent; Sc arising with 3/5 costa; R_2_ and R_3_ originating at corner of accessory cell; R_4_ and R_5_ fused and originating at apical corner of anterior part of the discoidal cell reaching to the apex; M_1_ and M_2_ parallel; M_2_ and M_3_ stalked at apical corner of posterior part of the discoidal cell; Cu_1_ and Cu_2_ parallel. Hindwing (Fig. 28): costa straight; discoidal cell 0.51 times as long as hindwing; Sc straight to 4/5 costa; R terminating at apex; M_1_ and M_2_ parallel, M_2_ and M_3_ originating at apical corner of posterior part of the discoidal cell (Table 2); Cu_1_ and Cu_2_ parallel to tornus. Legs: epiphysis absent (Fig. 9); femora and tibiae covered with brown hairs; tarsi covered with grayish brown scales.

***Female*** (Figs 24–27). Adult 4.2 mm in length. Coloration: Head, meso-, and metanotum dark brown. Membranous areas of abdomen yellowish brown. Abdomen covered with light brown scales; corethrogyne densely covered with white hairs on ventral part only. Structure: apterous. Head and compound eyes small, antennae slightly developed with basal flagellomeres 17 segmented, bipectinated. Legs well developed with tarsi divided into four tarsomeres; hind legs present with apical spurs.

***Male genitalia*** (Figs 11, 12). In lateral aspect. Genitalia index, 1.46–1.56 (Table 2). Dorsum gently curved. Saccus very short; ampulla narrow and short with club shape, setae present sparsely; harpe short with hooked shape; phallus slender and very long with whip shape. In dorso-ventral aspect, uncus slightly concave; gnathos and juxta absent; valva slightly narrow, apical part of valva densely covered.

***Female genitalia*** (Fig. 27). Oviscapt and ostium bursae well sclerotized. Apophyses posteriores 1.75 times longer than apophyses anteriores, very slender. Sclerotizations of the seventh sternite present with bundle of hairs

***Larval case*** (Figs 32, 33). Length 4.0 mm. Larvae build their cases by putting together small sand particles, forming oval-shaped cases rather than angular cases.

#### Distribution.

Korea.

#### DNA barcode.

DNA barcode sequences were generated from three individuals. Multiple alignments using the BLAST tool in the NCBI database showed the following species as nearest neighbor: *Dahlica charlottae* with a similarity between 97 and 95%.

#### Etymology.

The species is named in honor of Ms. Da-Som Kim, collector of the material.

### Dahlica (Dahlica) ochrostigma

Taxon classificationAnimaliaORDOFAMILIA

Roh & Byun sp. n.

http://zoobank.org/EAAEF10F-24B1-4FD8-A7A8-4C9313E4648C

Figs 13–19 , 20–23 , 29 , 34 , 35

#### Type material.

***Holotype.*** ♂ **Korea**: Gangwon-do, Taebaek-si, Changjuk-dong, 6.iii.2015, S.J. Roh & J.H. Jeon & T.H. Yoo, genitalia mounted on 80% glycerol solution, genitalia No. KNAESJ04, scales of forewing mounted on 80% glycerol solution, scales of forewing No. KNAESSJ03. venation of forewing No. KNAEVSJ03, Deposited at SEL/HNU.

***Paratypes.*** 7♂, 3♀. **Korea**: 3♂, 2♀, Gangwon-do, Pyeongchang-gun, Nodong-ri, 6.iii.2015, S.J. Roh & J.H. Jeon & T.H. Yoo, male genitalia mounted in 80% glycerol solution, genitalia No. KNAESJ05, scales of forewing mounted in 80% glycerol solution, scales of forewing No. KNAESSJ04, venation of forewing No. KNAEVSJ04, DNA barcode accession No. of male MF508657, DNA barcode accession No. of female MF664101; 4♂, 1♀ Gangwon-do, Taebaek-si, Changjuk-dong, GW, 6.iii.2015, S.J. Roh & J.H. Jeon & T.H. Yoo, scales of forewing mounted in 80% glycerol solution, scales of forewing No. KNAESSJ05, DNAbarcode accession No. of female MF664102. Deposited at SEL/HNU. Other material. 1♂ Korea: Gyeonggi-do, Paju-si, 2.iv.2007, B.W. Lee, genitalia mounted in 80% glycerol solution, genitalia No. KNAESJ06. Deposited at KNAE.

#### Diagnosis.

Male, this species is superficially similar to *D. somae* sp. n., but can be distinguished by slightly shorter antennae, a narrow forewing, and the venation of hindwing M3 stalked at 1/4 of M2. This species can be readily differentiated by the dorsum of male genitalia, which is strongly arched to the apical part and in the shape of a hat, and a very short phallus (lateral aspect). Female, apophyses posteriores 1.16 times longer than apophyses anteriores.

#### Description.

**Figures 13–19.**
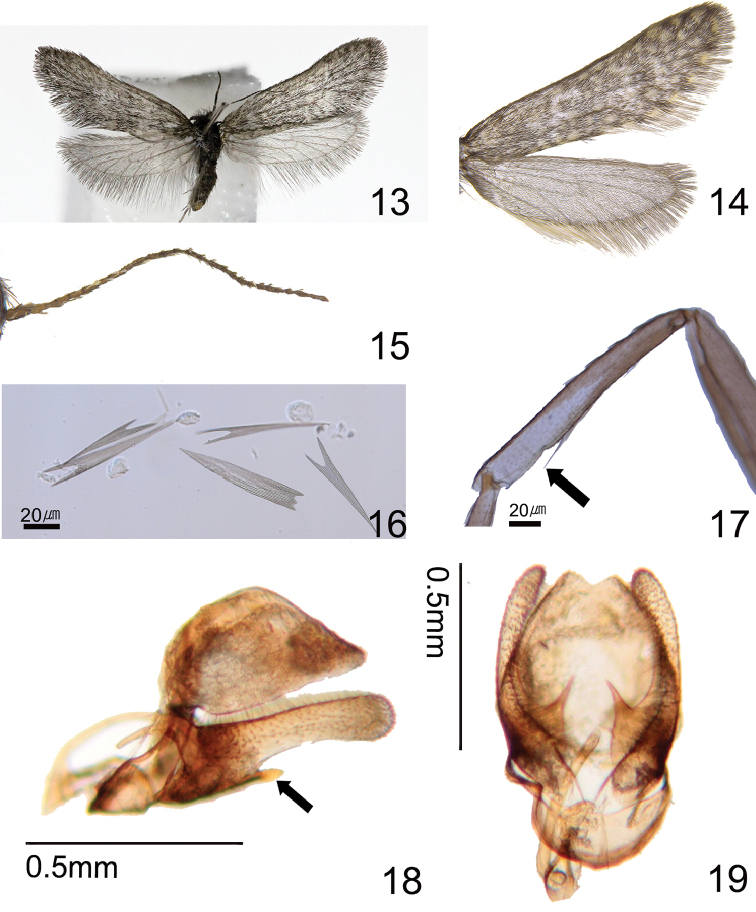
Male of Dahlica (Dahlica) ochrostigma. **13** Male, holotype **14** Close-up of rightwing-pattern, Paratype (Korea, Gangwon-do, Taebaek-si, Changjuk-dong, GW, 6.iii.2015) **15** Antenna, ditto **16** Scales of forewing, holotype (slide No. KNAESSJ03) **17** Absence of foreleg-tibia, holotype **18** Genitalia, holotype (slide No. KNAESJ04), lateral view **19** Ditto, dorso-ventral view.

Adult. Male (Figs 13–19). Wingspan 9.8–11.2 mm (Table 2). Coloration and vestiture: Vertex of head roughly covered with short grayish brown hairs. Thoracic notum covered with brown hairs. Upper side of forewing: ground color gray with sparsely yellow spots; scales (Fig. 16) considerably narrow; apical margin usually produced into two to three laciniations (classes 1–2) (Table 2). Postmarginal part of hindwing present with long shiny white hairs. Structure: head slightly small, compound eyes relatively large; ocelli absent. Antennae filiform (Fig. 15), less than 1/2 forewing. Forewing: short and narrow; costa straight; apex strongly arched to termen, discoidal cell 0.67 times as long as forewing; venation (Fig. 29) with nine veins, originating at the discoidal cell; intercalary cell absent and accessory cell present; Sc reaching to 3/5 costa; R_4_ and R_5_ fused; R_3_ and R_4_ + R_5_ originating at apical corner of anterior part of discoidal cell; M_1_ and M_2_ parallel; M_2_ and M_3_ stalked at apical corner of posterior part of the discoidal cell; Cu_1_ and Cu_2_ parallel. Hindwing (Fig. 29): costa straight; discoidal cell 0.52 times as long as hindwing; Sc straight and reaching to 4/5 costa; R originated at apical corner of anterior part of discoidal cell and reaching the apex; M_1_ and M_2_ parallel, M_3_ stalked at 1/4 M_2_ (Table 2); Cu_1_ and Cu_2_ parallel. Legs covered with shiny brown scales, epiphysis absent (Fig. 17).

**Figures 20–27.**
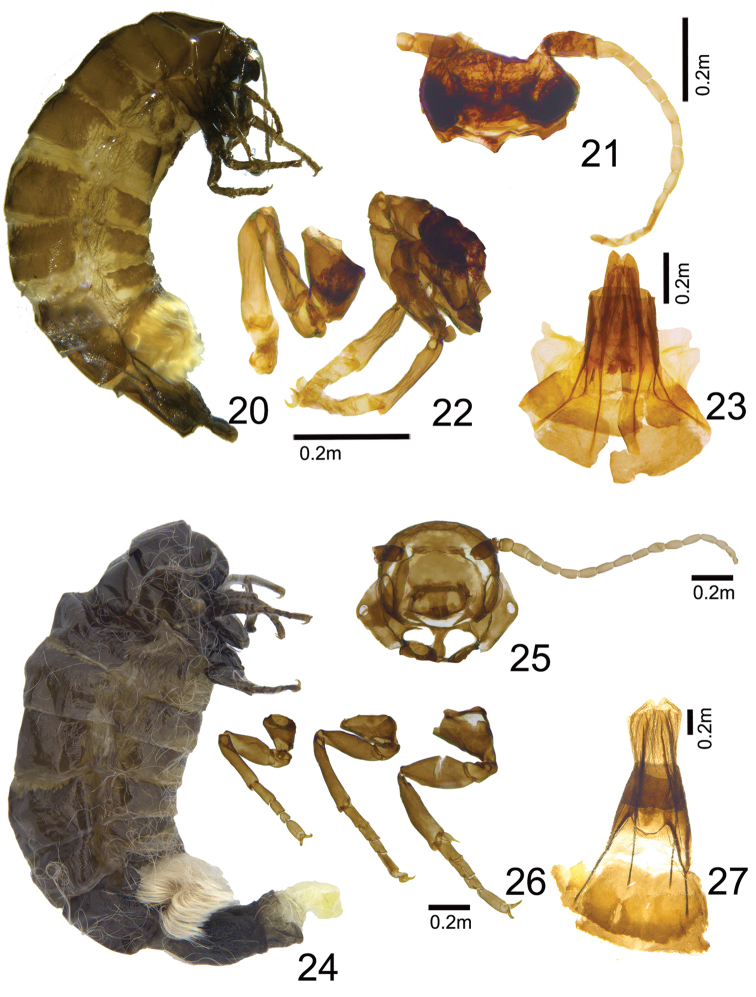
Females. **20** Dahlica (Dahlica) ochrostigma preserved in 70% ethanol, paratype (Korea, Gangwon-do, Taebaek-si, Changjuk-dong, GW, 6.iii.2015) **21** Ditto, head and antenna, macerated **22** Ditto, legs, foreleg to hindleg (left to right), macerated **23** Ditto, genitalia, macerated **24** Dahlica (Dahlica) somae, paratype (Korea, Daejeon, Mt. Heungnyongsan, 6.iii.2017) **25** Ditto, head and antenna, macerated **26** Ditto, legs, foreleg to hindleg (left to right), macerated **27** Ditto, genitalia, macerated.

***Female*** (Figs 20–23). 4.5 mm in length. Coloration: Head dark-brown. Meso and metanotum red-brown. Membranous areas of abdomen yellow. Abdomen clothed with light brown scales; corethrogyne densely covered with yellowish white hairs at only ventral part. Structure: Apterous. Head slightly small, antennae relatively developed and long. Legs well developed, slightly long, tarsi 4-segmented.

**Figures 28–29.**
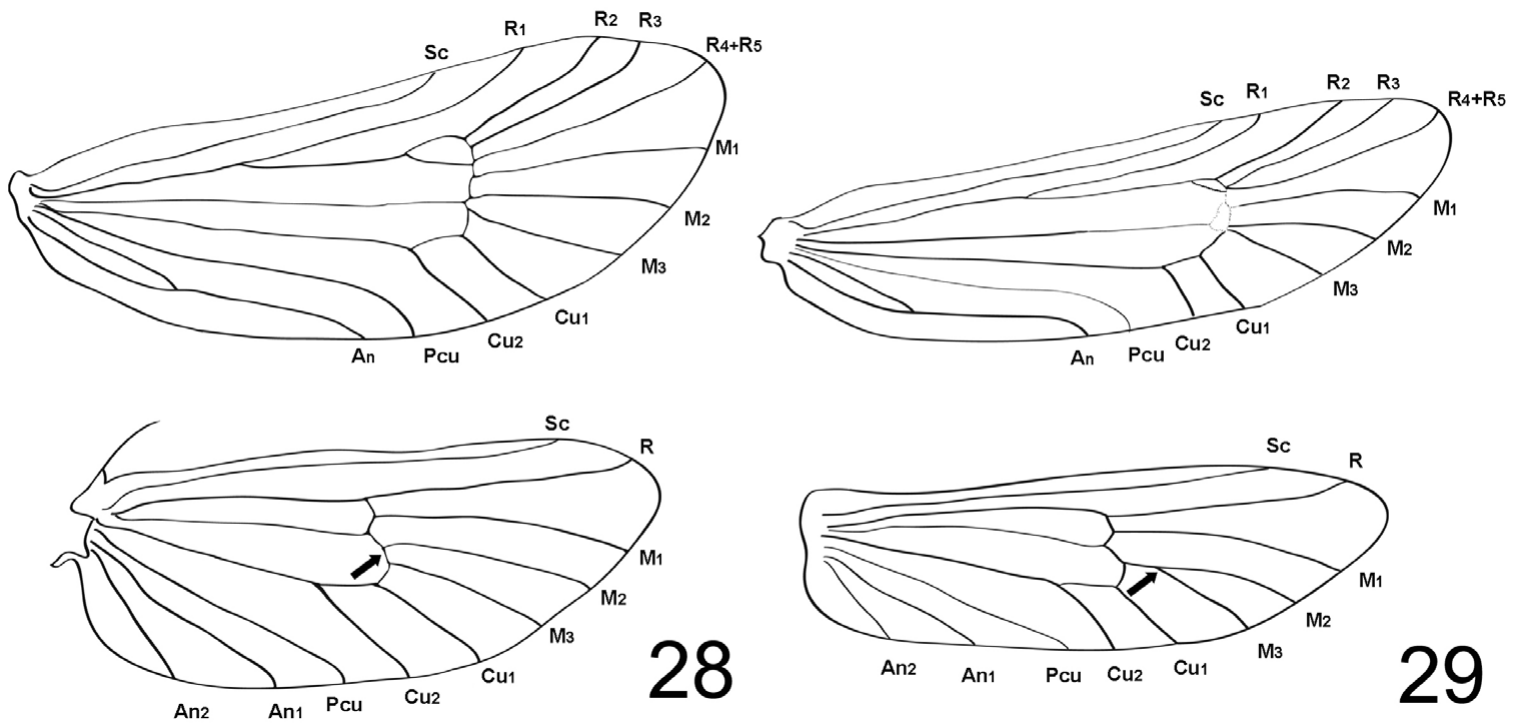
Wing venation of males. **28** Dahlica (Dahlica) somae, holotype (KNAEVSJ01) **29** Dahlica (Dahlica) ochrostigma, holotype (KNAEVSJ03).

***Male genitalia*** (Figs 18, 19). In lateral aspect. Genitalia index, 0.79–1.08 (Table 2). Dorsum strongly arched to apical part with the shape of a hat. Saccus relatively short; ampulla slightly long and club shape, setae sparsely; harpe short with needle shape; phallus slender and short with whip shape. In dorso-ventral aspect, uncus slightly concave shape; gnathos and juxta absent; valva slightly narrow and apical part produced into weak rounded claviform.

***Female genitalia*** (Fig. 23). Oviscapt and ostium bursae sclerotized. Apophyses posteriores 1.16 times longer than apophyses anteriores, slender. Sclerotizitions of seventh sternite present with bundle of hairs.

***Larval case*** (Figs 34, 35). Length 3.6-3.9 mm. Their cases are superficially similar to those of *D. somae* sp. n.

**Figures 30–35.**
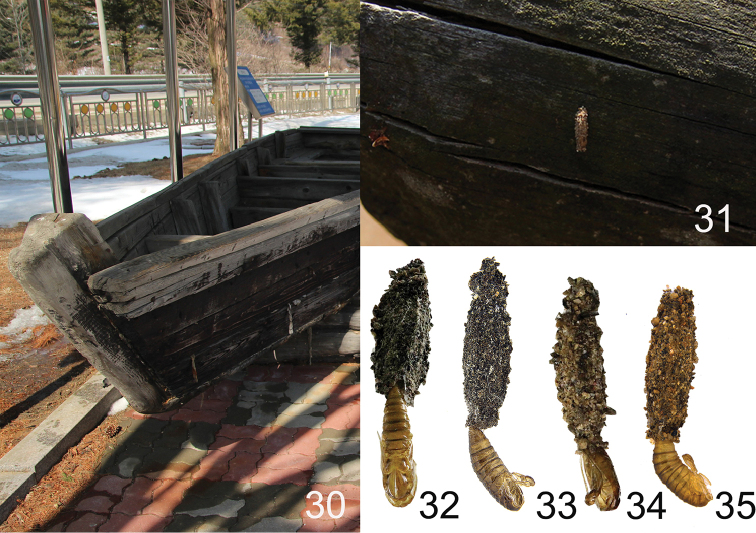
Microhabitat and larval cases with pupal exuviae. **30** Microhabitat of Dahlica (Dahlica) ochrostigma (Korea: Gangwon-do, Pyeongchang-gun, Nodong-ri, 6.iii.2015). **31** Ditto, close up **32** Male of Dahlica (Dahlica) somae, larval case with pupal exuviae **33** Female of D. (D.) somae, ditto **34** Male of D. (D.) ochrostigma, ditto **35** female of D. (D.) ochrostigma, ditto.

#### Distribution.

Korea.

#### DNA barcode.

DNA barcode sequences were generated from three individuals (Table 1). Multiple alignments using the BLAST tool in the NCBI database showed the following species as nearest neighbor, *Dahlica charlottae* with a similarity between 96 and 94%.

#### Etymology.

The specific name is derived from the Greek words *ochro* and *stigma* (= pale spots), referring to the forewing pattern.

## Discussion

**Figure 36.**
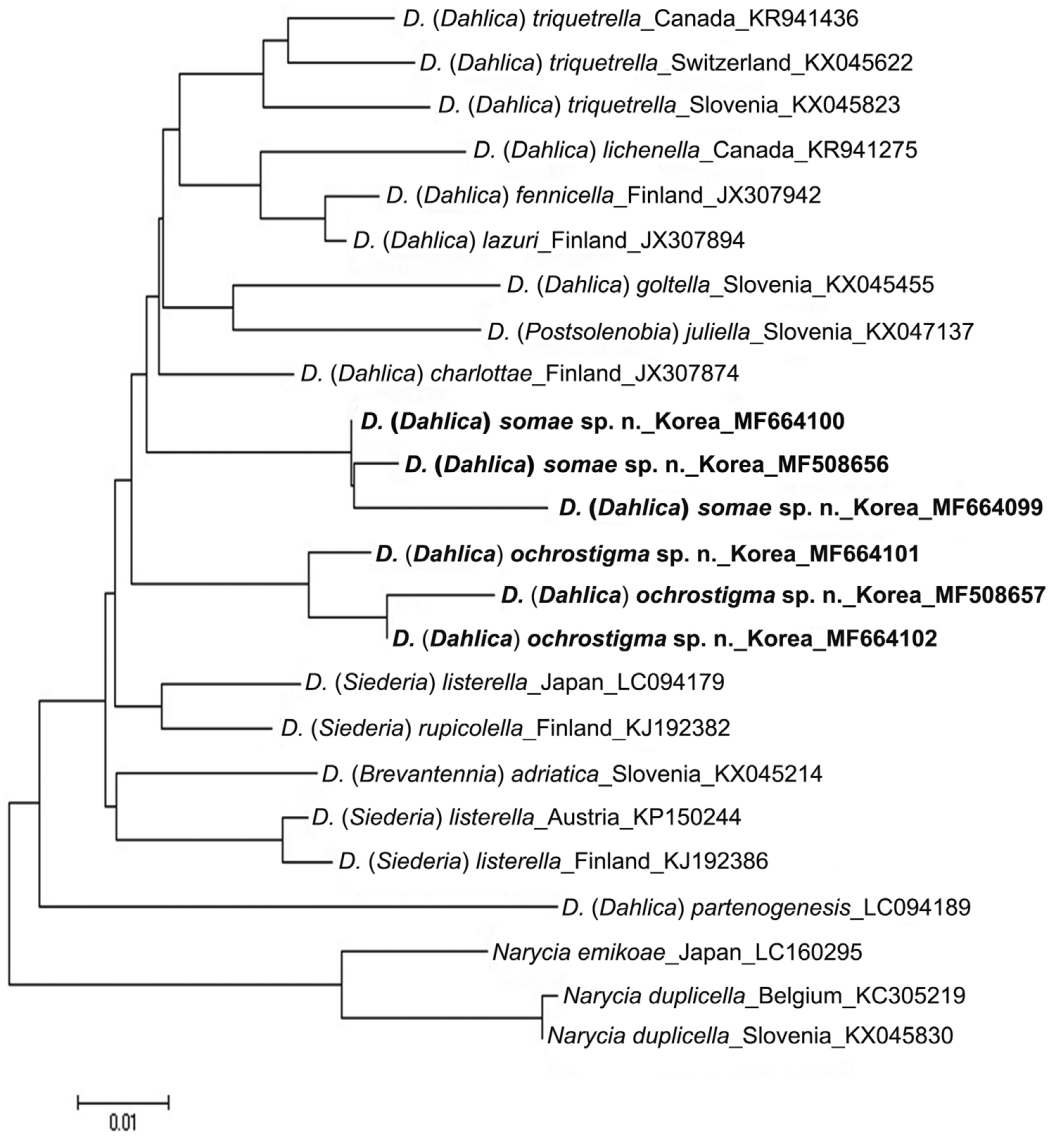
A Neighbor-joining tree, generated under the Kimura 2 parameter model (MEGA 6, Tamura et al. 2013) for the species of genera *Dahlica* and *Narycia* (DNA barcode data from NCBI). Branch lengths represent the number of substitutions per site as percentage.

The taxonomy of *Dahlica* has until recently been confusing owing to the similar morphology of the species in this genus and those in the allied genera *Siederia*, *Postsolenobia*, *Brevantennia*, and *Praesolenobia*. The proposal by Arnscheid and Weidlich (2017) to treat all these as subgenera of *Dahlica* has partly solved this problem. In this study, two new Korean species of *Dahlica* were reported for the first time with COI barcodes (Table 1). The results of comparison with related taxa, including subgenera of *Dahlica*, revealed no distinct differences (Fig. 36). Therefore, the taxonomic positions of the species in genus *Dahlica* needs to be redefined through future systematic studies with additional samples.

Most species of the genus *Dahlica* have been reported from Europe (48 species) to date, only one species, D. (D.) parthenogenesis Saigusa, 1961 was collected in Japan (Saigusa 1961). Thus, the two new species described in this study represent the first records for continental East Asia and serve as important basic data for future research on this genus and allied taxa in Asia.

## Supplementary Material

XML Treatment for Dahlica

XML Treatment for Dahlica (Dahlica) somae

XML Treatment for Dahlica (Dahlica) ochrostigma

## References

[B1] Arnscheid WR Weidlich M (2017) Microlepidoptera of Europe; Vol. 8 Psychidae. Brill, Leiden, 423 pp.

[B2] Arnscheid WR (2016) A new species of genus *Dahlica* Enderlein, 1912 form the Pyrenees of Aragon (Province of Huesca) in Spain (Lepidoptera: Psychidae: Dahlicini). SHILAP Revista de Lepidopterologia 44(173): 39–43.

[B3] Davis DR Robinson GS (1998) The Tineoidea and Gracillarioidea. In: Kristensen NP (Ed.) Lepidoptera, Moths and Butterflies. Vol 1: Evolution, Systematics and Biogeography. Handbook of Zoology 4. Walter de Gruyter, Berlin and New York, 91–117. https://doi.org/10.1515/9783110804744.91

[B4] Dierl W (1964) Cytologie, Morphologie und Anatomie der Sackspinner *Fumea casta* (Pallas) und *crassionella* (Bruand) sowie *Bruandia comitella* (Bruand) (Lepidoptera, Psychidae) mit Kreuzungsversuchen zur Klärung der Artspezifität. Zoologische Jahrbucher Systematik 91: 201–270.

[B5] Elzinga JA Jokela J Shama LNS (2013) Large variation in mitochondrial DNA of sexual and parthenogenetic *Dahlica triquetrella* (Lepidoptera: Psychidae) shows multiple origins of parthenogenesis. BMC Evolutionary Biology 13: 90. https://doi.org/10.1186/1471-2148-13-90 10.1186/1471-2148-13-90 PMC3655047 23622052

[B6] Enderlein G (1912) I. Wissenschaftliche Mitteilungen, 2. Zur Kenntnis der Zygophtalmen. Zoologischer Anzeiger 40: 261–282.

[B7] Grapputo A Kumpulainen T Mappes J (2005) Phylogeny and evolution of parthenogenesis in Finnish bagworm moth species (Lepidoptera: Psychidae: Naryciinae) based on mtDNA-marker. Annales Zoologici Fennici 42: 141–160.

[B8] Hebert DN Penton EH Burns JM Janzen DH Hallwachs W (2004) Ten species in one: DNA barcoding reveals cryptic species in the neotropical skipper butterfly Astraptes fulgerator. Proceedings of the National Academy of Sciences of the United States of America 101: 14812–14817. https://doi.org/10.1073/pnas.0406166101 15465915 10.1073/pnas.0406166101 PMC522015

[B9] Herrmann R (1988) *Dahlica marmorella* sp. n. eine neue Psychidae aus Italien (Lepidoptera: Psychidae). Nota Lepidopterologica 10(4): 203–208.

[B10] Herrmann R Weidlich M (1999) Psychidenbeobachtungen in Westrumänien.-Teil 2. Beschreibung von *Siederia transsylvanica* sp. n. (Psychidae). Nota Lepidopterologica 22(1): 10–16.

[B11] Hübner J (1796-1836) Sammlung europäischer Schmetterlinge. 8. Horde. Die Schaben; nach der Natur geordnet, beschrieben und vorgestellt. Augsburg, 1–78, pls 1–71.

[B12] Hori H (1926) A new Psychid from Japan. Kontyû 1: 28–30.

[B13] Katoh K Toh H (2008) Recent developments in the MAFFT multiple sequence alignment program. Briefings in Bioinformatics 9(4): 286–298. https://doi.org/10.1093/bib/bbn013 18372315 10.1093/bib/bbn013

[B14] Kristensen NP (2003) Skeleton and muscles: adults. In: Kristensen NP (Ed.) Lepidoptera, Moths and Butterflies, 2. Morphology, physiology and development. De Gruyter, Berlin, New York. Handbuch der Zoologie/ Handbook of Zoology 4(36), 39–131. https://doi.org/10.1515/9783110893724.39

[B15] Kumpulainen T (2004) The evolution and maintenance of reproductive strategies in bag worm moth (Lepidoptera: Psychidae). Jyväskylä Studies in Biological and Environmental Sciences 132: 1–42.

[B16] Kumpulainen T Grapputo A Mappes J (2004) Parasites and sexual reproduction in psychid moths. Evolution 58: 1511–1520. https://doi.org/10.1111/j.0014-3820.2004.tb01731.x 15341153 10.1111/j.0014-3820.2004.tb01731.x

[B17] Meier H (1958) Der taxonomische Wert der Hinterflügel-Aderung bei den Gattungen *Brevantennia* Sieder und Solenobia Duponchel (Lep., Psych.). Mitteilungen des naturwissenschaftlichen Vereins für Steiermark 88: 178–192.

[B18] Meyrick E (1935) Exotic Microlepidoptera, Vol. 4. Taylor and Francis, London, 577–608.

[B19] Mutanen M Wahlberg N Kaila L (2010) Comprehensive gene and taxon coverage elucidates radiation patterns in moths and bufferflies. Proceedings of the Royal Society B Biological Sciences 277: 2839–2848. https://doi.org/10.1098/rspb.2010.0392 20444718 10.1098/rspb.2010.0392 PMC2981981

[B20] Nieukerken EJ van Kaila L Kitching IJ Kristensen NP Lees DC Minet J Mitter C Mutanen M Regier JC Simonsen TJ Wahlberg N Yen SH Zahiri R Adamski D Baixeras J Bartsch D Bengtsson BA Brown JW Bucheli SR Davis DR De Prins J De Prins W Epstein ME Gentili-Poole P Gielis C Hattenschwiler P Hausmann A Holloway JD Kallies A Karsholt O Kawahara AY Koster SJC Kozlov MV Lafontaine JD Lamas G Landry JF Lee S Nuss M Park KT Penz C Rota J Schitlmeister A Schmidt BC Sohn JC Solis MA Tarmann GM Warren AD Weller S Yakovlev RV Zolotuhin VV Zwick A (2011) Order Lepidoptera Linnaeus, 1758. In: Zhang ZQ (Ed.) Animal biodiversity: an outline of higher-level classification and survey of taxonomic richness. Zootaxa 3148: 212–221.

[B21] Rekelj J Predovnik Ž (2014) *Dahlica goltella* sp. n., a new bagworm species from Slovenia (Lepidoptera: Psychidae). Acta Entomologica Slovenica 22(1): 5–18.

[B22] Regier JC Mitter C Davis DR Harrison TL Sohn JC Cummings MP Zwick A Mitter KT (2015) A molecular phylogeny and revised classification for the oldest ditrysian moth lineages (Lepidoptera: Tineoidea), with implications for ancestral feeding habits of the mega-diverse Ditrysia. Systematic Entomology 40: 409–432. https://doi.org/10.1111/syen.12110

[B23] Roh SJ Byun BK (2017a) First discovery of the Lichen-Feeding Moth *Bacotia sakabei* (Lepidoptera: Psychidae) from Korea. Animal Systematics, Evolution and Diversity 33(1): 60–64. https://doi.org/10.5635/ASED.2017.33.1.064

[B24] Roh SJ Byun BK (2017b) Two species of the subfamily Psychinae (Lepidoptera: Psychidae) new to Korea. Journal of Asia-Pacific Biodiversity 10(2): 224–227. https://doi.org/10.1016/j.japb.2017.04.014

[B25] Roh SJ Banasiak G Byun BK (2016) A new and an unrecorded species of the family Psychidae (Lepidoptera) from Korea, with an annotated catalogue. Journal of Natural History 50(11/12): 669–680. https://doi.org/10.1080/00222933.2015.1082654

[B26] Roh SJ Byun BK (2016) Discovery of *Ceratosticha leptodeta* Meyrick (Lepidoptera: Psychidae) from Korea. Journal of Asia-Pacific Biodiversity 9(1): 91–93. https://doi.org/10.1016/j.japb.2015.12.009

[B27] Saigusa T (1961) Systematic studies of *Diplodoma* and its allied genera in Japan. Sieboldia. II/ 4: 261–315.

[B28] Saigusa T Sugimoto M (2014) Japanese species of the genus *Proutia* Tutt, 1899 (Lepidoptera: Psychidae). Zootaxa 3869: 143–152. https://doi.org/10.11646/zootaxa.3869.2.3 25283906 10.11646/zootaxa.3869.2.3

[B29] Sauter W (1956) Morphologie und Systematik der schweizerischen *Solenobia*- Arten. Revue Suisse de Zoologie 63: 451–550. https://doi.org/10.5962/bhl.part.75469

[B30] Sauter W Hättenschwiler P (1991) Zum System der palaearktischen Psychiden (Psychidae) 1 Teil: Liste der paläarktischen Arten. Nota lepidopterologica 22: 262–295.

[B31] Sauter W Hättenschwiler P (1999) Zum System der palaearktischen Psychiden (Psychidae) 2 Teil: Bestimmungsschlüssel für die Gattungen. Nota lepidopterologica 22: 262–295.

[B32] Seino A (1981) A new psychid species of *Bacotia* from Japan (Lepidoptera). Tyô to Ga 31: 121–125.

[B33] Sieder L (1953) Vorerbeit zu einer Monographie über sie Gattung Solenobia Z. (Lepidopt. Psychidae-Taleporiinae). Zeitschrift der Wiener Entomologischen Gesellschaft 38(5): 113–128.

[B34] Sobczyk T (2011) World catalogue of insects; Vol.10 Psychidae(Lepidoptera). Apollo Books, Stenstrup, 467 pp.

[B35] Sugimoto M (2009a) A comparative study of larval cases of Japanese Psychidae(Lepidoptera). Japanese Journal of Entomology (NS) 12: 1–15.

[B36] Sugimoto M (2009b) A comparative study of larval cases of Japanese Psychidae(Lepidoptera). Japanese Journal of Entomology (NS) 12: 17–29.

[B37] Suomalainen E (1980) The Solenobiinae species of Finland (Lepidoptera: Psychidae), with description of a new species. Entomologica Scandinavica 11: 458–466. https://doi.org/10.1163/187631280794710042

[B38] Tamura K Stecher G Peterson D Filipski A Kumar S (2013) MEGA6: Molecular Evolutionary Genetics Analysis version 6.0. Molecular Biology and Evolution 30: 2725–2729. https://doi.org/10.1093/molbev/mst197 24132122 10.1093/molbev/mst197 PMC3840312

